# Characteristics of drug-facilitated sexual assault (DFSA): an observational study at a Danish sexual assault center

**DOI:** 10.1007/s00414-025-03646-4

**Published:** 2025-11-04

**Authors:** Marina Rasmussen, Johannes Rødbro Busch, Julie Munkholm, Kathrine Østerballe Skov, Marie Katrine Klose Nielsen, Malene Hilden, Jytte Banner, Carl Johan Wingren

**Affiliations:** 1 Department of Forensic Medicine, Section of Forensic Pathology, University of Copenhagen, Frederik V’s Vej 11, Copenhagen, 2100 Denmark; 2 Department of Forensic Medicine, Section of Forensic Chemistry, University of Copenhagen, Frederik V’s Vej 11, Copenhagen, 2100 Denmark; 3 Department of Gynecology, Fertility and Obstetrics, Copenhagen University Hospital, Blegdamsvej 9, Copenhagen, 2100 Denmark

**Keywords:** Drug-facilitated sexual assault (DFSA), Sexual violence, Forensic toxicology, Clinical forensic medicine, Injuries, Genital injuries

## Abstract

**Introduction:**

In drug facilitated sexual assault (DFSA) the victim is unable to consent to the sexual act due to influence of alcohol, narcotics, or medicine. Increasing the awareness of characteristics associated with DFSA can assist the judicial system in early recognition of cases and guide public health interventions for reducing DFSA. This study aims to assess characteristics of DFSA victims compared to victims of non-DFSA.

**Methods:**

We included 643 females from the age of 15, examined at Center for Victims of Sexual Assault (CVSA), Copenhagen, Denmark from 2021 to 2023. We extracted information on context of the case, findings of the examination, and linked register data, including socioeconomics, criminal records, and prescribed medication. We applied descriptive statistics and logistic regression models.

**Results:**

DFSA victims, compared to non-DFSA, were younger and the risk was higher during weekends, at night, and by a perpetrator met for the first time the day of the assault. DFSA was associated with occurring at a public place, or the home of a friend. More injuries were observed in cases examined within 24 h of the assault. Ethanol, cocaine, and THC were the most frequent substances. Victims of sexual assault under 18 years have more reports to child protection services compared to the general population.

**Conclusion:**

In inebriated, young victims with memory loss, stating an assault at a public place the concern for DFSA should be raised. Early in the investigation of sexual assaults, forensic examination should be prioritized for securing samples for toxicology and documenting injuries.

## Introduction

Drug facilitated sexual assault (DFSA) is defined as a sexual assault in which the victim is unable to consent to the sexual act due to the influence of alcohol, narcotics, or medicine. Previous studies have shown that in most cases a perpetrator exploits a victim´s impaired state due to their voluntary consumption of alcohol or recreational drugs, a so-called opportunistic assault. Less frequently, the perpetrator actively drugs the victim, either forcibly or covertly, leading to what is termed a proactive or predatory assault [1].

There is no definitive list of substances associated with DFSA, as any drug that lowers level of consciousness, impairs memory functions, or affects psychomotor control are potential candidates. However, retrospective data has shown that alcohol, followed by benzodiazepines and cocaine, are the most frequently observed substances associated with DFSA [1–5].

The prevalence of sexual assault is difficult to establish due to a possible large hidden number, and this is probably also the case for DFSA. Based on a nationwide, anonymous survey conducted from 2005 to 2024 by The Danish Department of Justice, an estimated 12.000 individuals in the Danish population reported being victims of sexual assault or attempted sexual assault within the last year [6]. Approximately half of the victims stated that they were under the influence of either alcohol or an illicit drug during the assault. In similar surveys from other countries, up to two-thirds of all victims of sexual assaults report being alcohol inebriated during the assault [7–10] and DFSA victims are more likely to have consumed any amount of alcohol compared to non-DFSA victims [11].

It is important to distinguish between DFSA and non-DFSA in both a research context and an investigative context. Drugging can affect the victims’ memory, behavior, and psychomotor control, which in turn may lead to feelings of uncertainty, of guilt, and fear of being stigmatized. Furthermore, a side effect of drugging is sedation, which could lead to a delay in contacting a sexual assault center and thereby challenges in detecting the drug in blood and/or urine. Knowledge about characteristics associated with DFSA can help identify risk factors and inform targeted public health efforts for reducing DFSA. However, a recent review have identified gaps in knowledge about potential risk factors associated with DFSA, e.g. the categorization of DFSA [12].

We aim to assess characteristics of DFSA victims in comparison to victims of non-DFSA in a cohort of victims referred for a clinical forensic exam at a Danish sexual assault center. We study various characteristics of the victims, including demographics, socioeconomics, mental health, previous exposure to crime, toxicology and injuries, as well as associations with the perpetrator and crime scene characteristics.

## Methods and material

### Design and study population

We identified victims aged 15 years and older that were examined at the Center for Victims of Sexual Assault (CVSA), Copenhagen, within 7 days after the sexual assault between Jan 1 st 2021 and Dec 31 st 2023.We used data extracted from written reports based on clinical forensic examinations of victims performed at the CVSA. Reporting the sexual assault to the police is not a prerequisite for undergoing an examination at the sexual assault center; victims may choose to file a police report later.

**Fig. 1:**
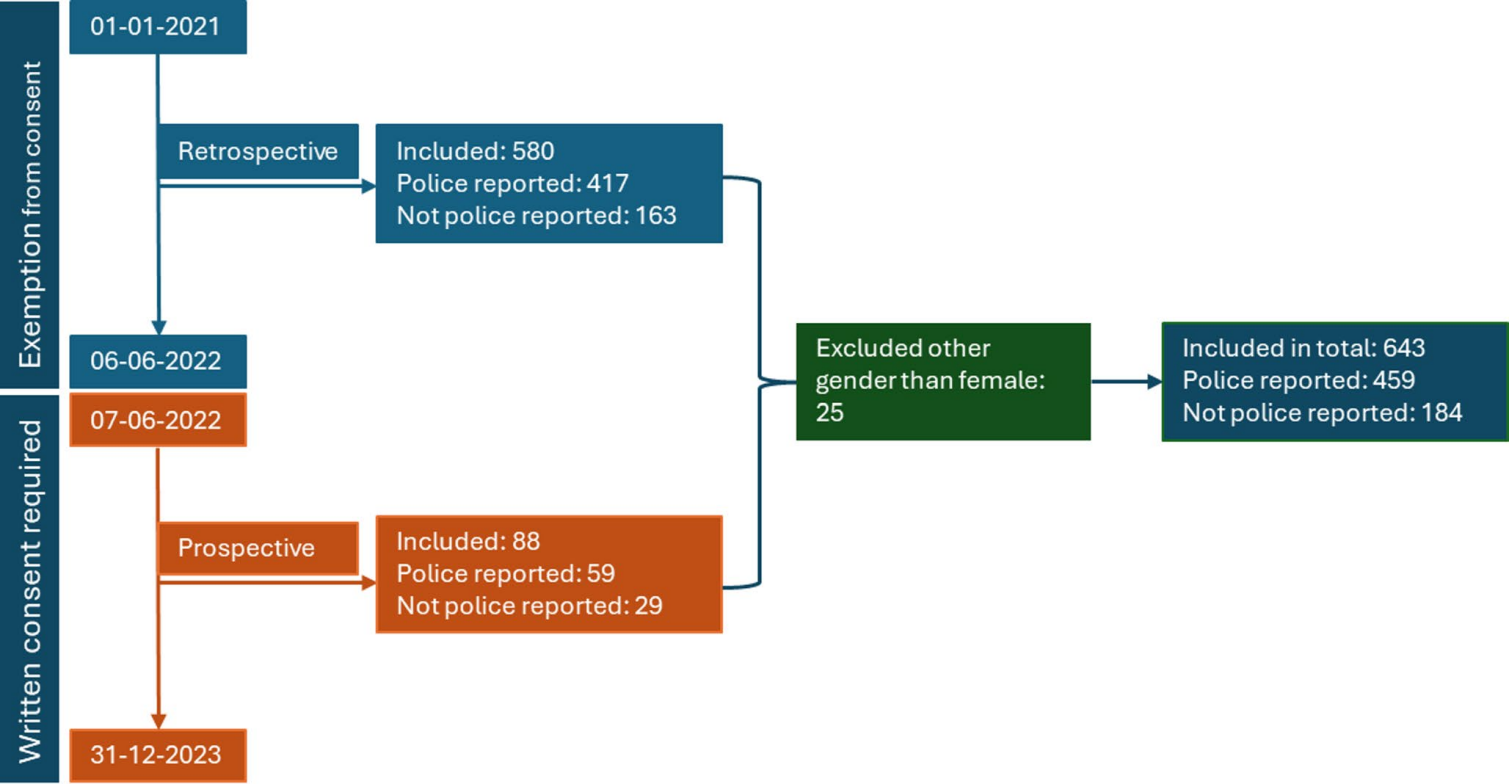
Illustration of the study periods and how the study population is distributed between a retrospective and a prospective part and how many of the assaults that were reported to the police

The study population consisted of a series of retrospectively included cases and an additional series of prospectively included cases (Fig. 1). We obtained ethical approval to study all cases that were examined from Jan 1 st 2021 to Jun 6th 2022 (*n* = 580). The ethical approval included an exemption of consent to analyze blood and urine samples. Hence, a clinical biobank of blood and urine samples collected at forensic medical examinations from the preceding 18 months was available for toxicological analysis. If toxicological analysis had been performed as part of the police investigation of the case, we retrieved the results from the toxicology reports.

The second half of the study period was between June 7th 2022 and Dec 31 st 2023 and included prospectively gathered data. In this period, it was mandatory to ask all victims for written consent to have their blood and urine samples analyzed as part of this research project (*n* = 88). The total study population was thus 668 cases.

Due to the low number of male and transgender cases we excluded genders other than females (*n* = 25), leaving 643 cases for analysis.

### Examination at the center for victims of sexual assault (CVSA)

The CVSA covers the entire area of Eastern Denmark with a population of about 2.5 million people. If they contact the CVSA within 7 days of the alleged assault, victims are offered an acute, clinical forensic examination consisting of a full body examination including a gynecological examination and an anal examination in relevant cases, collection of DNA traces, and obtaining blood and urine samples for possible later toxicological analysis. Examinations at the CVSA are performed either by a forensic physician or a specially trained gynecologist using the same standardized procedure. An exam requires neither a referral nor a police report. Samples are kept for 12 months. If the victim decides to press charges after the initial examination within 12 months, the evidence is turned over to the police. Such “late reports” occur in 7,9% of the cases not reported to the police at the time of the examination [13].In conjunction with the examination, the victim is offered routine screening for venereal diseases, a pregnancy test, and consultations with a psychologist. “If the victims present more than 7 days after the sexual assault the police can choose to order a clinical forensic examination, but in these cases the main focus is on documenting injuries, and in turn collection of DNA traces and blood and urine samples are left out. Hair samples for toxicological analysis can however still be obtained after at least 4–6 weeks in relevant cases.”

In addition to securing forensic evidence at the examination, the victim is interviewed by the physician regarding the details of the assault, including their relation to the perpetrator, time, and location of the crime, as well as the victim’s physical and mental health. A standardized form is utilized, meaning anamnestic data is collected in a structured manner [14, 15].

### Data extraction and DFSA case definition

**Fig. 2:**
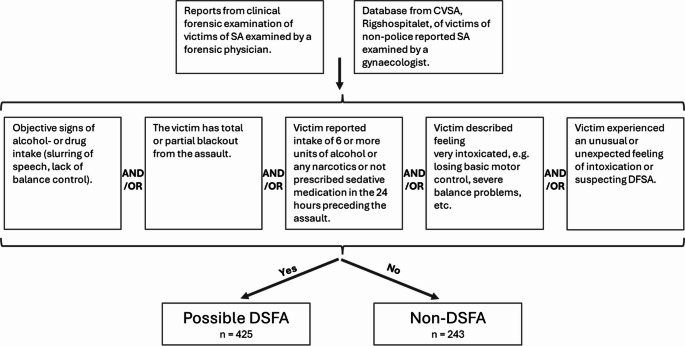
This figure shows the criteria used for categorizing the case to either a possible DFSA or non-DFSA

We performed manual data extraction from forensic reports to obtain information on criminological and interpersonal characteristics. Data was extracted using a predefined template and registered in a database. Concomitantly, we categorized the assault as either a possible DFSA or non-DFSA, based on five standardized criteria of which at least one criteria should be fulfilled to be included as a DFSA-case (Fig. 2). To use an example, if a victim objectively appeared affected by alcohol and/or drugs the case was categorized as a possible DFSA. If the victim did not appear affected but stated that they had been drinking more than 5 units of alcohol or taken any illicit drug or sedative medication within 24 h prior to the sexual assault the cases is also categorized as a DFSA. If the victim stated to have partial or total blackout from the sexual assault or felt very intoxicated or had an unusual feeling of intoxication the case was categorized as DFSA. An example of an unusual feeling of intoxication could be if the victim only had one drink but felt very intoxicated by losing basic motor control. In this study, we did not separate between proactive and opportunistic DFSA. Data related to cases not reported to the police was not summarized in written reports and was only accessible in a clinical research database. We merged the data from the clinical research database with the forensic research database, using predefined merging algorithms to ensure homogeneity of variables.

Two authors validated the classification of DFSA and non-DFSA in 131 (29%) of the police-reported cases and 72 (39%) of the non-police-reported cases. Initially, there was a disagreement in 5% of the police-reported cases, after which the criteria were reformulated. Following this reformulation, there was 100% agreement.

### Toxicological analysis

Blood and urine samples were collected during forensic medical examinations that occurred up to 7 days after the assault. Samples were collected in vacutainers with sodium fluoride and potassium oxalate and stored at − 20 °C prior to analysis. Analysis was performed at the Section of Forensic Chemistry, Department of Forensic Medicine, University of Copenhagen.

Of the 668 cases, blood or urine samples were not available in 7 cases. In 128 cases, samples had already been analysed for ethanol, illicit drugs, and pharmaceuticals, on request by the police as an investigative measure in the individual case handling [16]. The results of these analyses were extracted from a database. In 533 cases, blood and urine samples were analyzed for DFSA relevant substances using a systematic toxicological analysis strategy described by Skov et al. [17]. Urine samples were prepared using an automated sample preparation including enzymatic hydrolysis and protein precipitation on a Hamilton Microlab Vantage 2.0 liquid handling system (Hamilton Company, Bonaduz, Switzerland). Toxicological analysis included initial screening by ultra-high pressure liquid chromatography–tandem mass spectrometry (UHPLC–MS/MS) using a comprehensive multi-target method [17]. Positive screening results were verified in whole blood using UHPLC–MS/MS. Both screening and verification were performed using ACQUITY UHPLC Systems coupled to Xevo TQ-S Detectors (Waters, Milford, MA, USA). The limits of detection used during analysis were in accordance with guidelines from The Society of Forensic Toxicologists (SOFT) [18].

Ethanol screening was conducted in cases where samples were collected within 24 h of the assault. Analysis for ethanol was performed in blood samples from 332 cases by headspace gas chromatography with flame ionization (HS-GC-FID) [16].

## Description of study variables

### Data obtained from clinical examinations of the victims

Age was categorized into (i) 15–19 years, (ii) 20–24 years, (iii) 25–29 years, (iv) 30–34 years, (v) 35–39 years and > 39 years. We applied the age group (iii) 25–29 years as reference in the regression analysis. For stratified analyses we also categorized cases into an age-group (15–24 years), and an age-group (> 24 years).

The day of the sexual assault was categorized into (i) weekday (Monday to Thursday), (ii) weekend (Friday to Sunday), or (iii) not specified, and the time was categorized into (i) 00:00 to 05:59, (ii) 06:00 to 11:59, (iii) 12:00 to 17:59, (iv) 18:00 to 23:59 and (v) not specified. For regression analysis with day of the week we applied category (i) weekday as reference, and in analysis of time of day we applied category (i) 00:00 to 05:59 as reference. Time from the sexual assault to examination was categorized as (i) hours (up to 24 h from the sexual assault to the examination), (ii) days (more than 24 h and up to 7 days from the sexual assault to the examination) and (iii) not specified. In the regression analysis we applied category (i) hours as reference.

The relation between the victim and the perpetrator were defined as (i) partner or ex-partner, (ii) acquaintance, defined as someone the victim knew peripherally, (iii) contact, defined as someone the victim met on the day of the sexual assault, (iv) virtual contact, defined as a contact established in an online forum with their first meeting at the day of the sexual, (v) not specified or no recollection, (vi) friend, (vii) miscellaneous relations, e.g. distant family, taxi driver, or colleague, and (viii) stranger. In the regression analysis, we applied the category (i) acquaintance as reference.

The scene of the crime was categorized as (i) perpetrator´s home, (ii) a third party´s home, (iii) the victim’s home, (iv) outdoor location (street/park/parking lot, etc.), (v) club or bar, (vi) no knowledge/recollection, or unspecified, or (vii) other, defined as hotel, car, vacation house, or public building. In the regression analysis we applied category (i) perpetrator’s home as reference.

Victims reporting either physical violence, penile penetration, vaginal penetration, oral penetration, or anal penetration were categorized into (i) no, (ii) yes, and (iii) no knowledge/recollection. In the regression analysis we applied (i) no as reference. Penile penetration was defined as any form of penile penetration, either vaginal, oral, and/or anal.

The presence of either extragenital and/or genital lesions were both categorized into no or yes. Extragenital lesions include injuries (abrasions, bruises, bite marks, lacerations etc.) on the body from head to toe excluding the anogenital area. Anogenital lesions include injuries on the external genitalia, hymen, vagina, perineum, and perianal.

### Data obtained from national registries

We included national registry data obtained from Statistics Denmark and the Danish Health Registries.

As socioeconomic indicators we included education and income level. Education level was defined as the highest completed education at the time of the sexual assault and was categorized into (i) primary school (ii) high school, (iii) vocational education/short-cycle higher education, (iv) medium-cycle/long-cycle higher education or PhD, etc. and (v) missing. We included (i) primary school as reference in the analyses. Income level was dichotomized as living in a household with an income above or below the 50% threshold of the national median income. This level is considered the statistical definition of living in relative poverty in Denmark. We included low income as reference in the analysis.

Having at least one previous report to municipal child protective services (CPS) within the last 5 years prior to the sexual assault was categorized as no or yes. Such reports are made by citizens or professionals to express concern for the well-being of a child or adolescent. This may involve suspicion of neglect, violence, sexual abuse or other circumstances that may endanger the health or development of the child or adolescent. According to Danish legislation, everyone has a civic duty to report if they become aware of such circumstances, but child and health care professionals have a legal obligation to report any concerns for a child’s welfare. Five years prior to the study period is the current span of available registry data, thus we could not apply for data further back, meaning victims above the age of 23 at the date of assault would therefore be registered as negative, even if any prior reports may had been filed when they were younger. We included cases with no previous registered reports as references.

We extracted data from crime record archives on previous registrations as being a victim of violent crimes (either interpersonal violence or sexual assault) within the last 5 years prior to the sexual assault, the variable was dichotomized into yes or no. We included cases with no previous registrations as references.

We obtained information concerning if the victim had collected a prescribed medication at a pharmacy within 6 months before the sexual assault. We included only medication groups that we deemed relevant, i.e. have either stimulating or sedating CNS-effects: antidepressants, antiepileptics, antipsychotics, benzodiazepines, opioids, stimulants, and sedative-hypnotics. If an individual had picked up at least one prescription for a medication in these categories, their prescription status for this drug was categorized as yes, otherwise their prescription status was categorized as no.

### Data obtained from toxicological analysis

Substances identified in toxicological analysis were divided into three main categories, ethanol, illicit drugs (THC, cocaine, methamphetamine, amphetamine, GHB, MDMA) and medication (sedative antihistamines, antidepressants, antiepileptics, antipsychotics, benzodiazepines, opioids, stimulants, and sedative-hypnotics), and for each of these groups the analytical results were categorized as negative or positive. We included negative results as reference in the analysis.

## Statistical analysis

Analysis was performed using Statistical Package for the Social Sciences (SPSS^®^, version 29.0.2.0 (20) IBM^®^).

We performed univariate logistic regression analyses in which each of the characteristics were analyzed for association with DFSA in comparison to non-DFSA. Further, we performed univariate logistic regression analysis studying the association between injuries and time between the sexual assault and the examination. Also, we performed sensitivity analysis on the findings of drugs by restricting the analysis to the population examined within 24 h after the sexual assault. Furthermore, to better understand the association between age and the observed characteristics we performed sensitivity analyses in which the associations between characteristics and DFSA compared to non-DFSA were examined in strata of age categorized into (i) 15–24 years and (ii) > 24 years) and (i) < 40 years and (ii) ≥ 40 years.

All analyses were considered statistically significant with a *p-*value of less than 0.05 and we applied a 95% confidence interval (CI).

## Results

In total, we collected data from 668 cases of sexual assault during the 3-year period, of which 425 (63.6%) cases were categorized as possible DFSA, and 243 (35.9%) cases were categorized as non-DFSA. Two authors validated the classification with 0% disagreement.

As stated above, most of the victims (*n* = 643, 96.4%) were females, of which 63.1% were categorized as possible DFSA. Most cases where the victim was male were categorized as possible DFSA (85.7%). There were less than 5 transgender cases. In the regression analysis, we only included female victims, due to the low number of male and transgender cases, including a total of 643 female cases for further analysis.

### Victim characteristics

In the DFSA group, the age category 15–19 years was most frequent and for non-DFSA the category 20–24 years (Table 1). 55.4% of all victims of sexual assault had an examination performed within 24 h after the sexual assault. In 56% of victims below the age of 18 years, a child protection services report had been registered within the last 5 years prior to the sexual assault.

**Table 1:** Descriptives of victim characteristics and association with DFSA compared to non-DFSA in univariate logistic regression models

Characteristics	Non-DFSA (*n* (%))	DFSA (*n* (%))	OR	95% C.I. for OR		*p*-value
				Lower	Upper	
Total number	237 (36.9)	406 (63.1)				
Age group (years)						
25-29	29 (12.2)	65 (16.0)	*Ref.*			
15-19	59 (24.9)	136 (33.5)	1.03	0.60	1.75	0.92
20-24	74 (31.2)	123 (30.3)	0.74	0.44	1.25	0.26
30-34	25 (10.2)	34 (8.4)	0.61	0.31	1.19	0.15
35-39	15 (6.3)	18 (4.4)	0.54	0.24	1.21	0.13
>40	35 (14.8)	30 (7.3)	0.38	0.20	0.75	<0.01
Time from SA to examination						
<24 hours	125 (52.7)	231 (56.9)	*Ref.*			
>24 hours	107 (45.1)	165 (40.6)	0.83	0.60	1.16	0.28
NS	5 (2.1)	10 (2.5)	1.08	0.36	3.24	0.89
Education level						
Primary school	117 (49.4)	176 (43.3)	*Ref.*			
High school	45 (19.0)	105 (25.9)	1.56	1.02	2.36	0.04
Vocational education/short-cycle higher education	22 (9.3)	39 (9.6)	1.18	0.67	2.09	0.57
Medium-cycle/long-cycle higher education or PhD, etc.	28 (11.8)	54 (13.3)	1.28	0.77	2.14	0.34
Missing	25 (10.5)	32 (7.9)	0.85	0.48	1.51	0.58
Low-income 50 pct. (Lower income versus higher income)	78 (35.0) / 145 (65.3)	131 (34.0) / 254 (66.6)	0.96	0.68	1.36	0.81
Victim of other volent crime within the last 5 years (no/yes)	223 (94.1) / 14 (5.9)	379 (93.3) / 27 (6.7)	1.14	0.58	2.21	0.71
Prescribed medication* within the last 6 months prior to the SA (no/yes)	146 (67.3) / 71 (32.7)	279 (72.7) / 105 (27.3)	1.29	0.90	1.86	0.165

SA = sexual assault, NS = Not specified; the information is not specified in the available data

*Only medication that effects the CNS is accounted (ATC-code N), data was available in 601 cases (42 missing)

In a univariate logistic regression model, we observed that victims of sexual assault over the age of 40 years, were less likely victims of DFSA (OR: 0.38: 95% CI 0.20–0.75) (Table 1). Additionally, an indication of a trend of decreasing risk of DFSA with increasing age was observed. We observed no differences between the DFSA and non-DFSA victims in socioeconomic measures, including education and income level. Also, we observed no increased frequency in victims of DFSA to having been a victim of violent crimes in the 5 years preceding the assault (Table 1).

### Assault characteristics

Significantly more DFSAs occurred in the weekend (OR 2.18: 95% CI 1.54, 3.09), and from midnight to the morning, (Table 2). In DFSA cases, the perpetrator was more often a contact the victim met on the day of the assault (OR 2.63: 95% CI 1.58, 4.38), in contrast to in non-DFSA cases in which the perpetrator was more often a partner or an ex-partner. The scenes of the assault in DFSA cases were more frequently a club or bar (OR 5.63: 95% CI 1.88, 16.8), an outdoor location (OR 2.29, 95% CI 1.20, 4.39) or in the home of a friend (OR 1.94: 95% CI 1.15, 3.29). We also found that the perpetrator was described as a stranger (i.e. assault rape) in only a small number of all sexual assaults (4.4%), approximately similar for both DFSA (4.4%) and non-DFSA (3.8%) (Table 2). In DFSA cases, there were more often multiple perpetrators, compared to non-DFSA (OR 6.21: 95% CI 1.86, 20.78).

**Table 2:** Descriptives of assault characteristics and univariate logistic regression in DFSA compared to non-DFSA

Characteristics	Non-DFSA (*n* (%))	DFSA (*n* (%))	OR	95% C.I. for OR		*p*-value
				Lower	Upper	
Total number	237 (36.9)	406 (63.1)				
Day of the SA						
Weekday (Monday-Thursday)			*Ref.*			
Weekend (Friday-Sunday)			2.18	1.54	3.09	<0.01
NS			2.50	0.47	13.2	0.28
Time of the SA						
00:00 – 05:59	103 (43.5)	389 (71.2)	*Ref.*			
06:00 – 11:59	26 (11.0)	48 (11.8)	0.66	0.39	1.12	0.12
12:00 – 17:59	35 (14.8)	9 (2.2)	0.09	0.04	0.20	<0.01
18:00 – 23:59	55 (23.2)	42 (10.3)	0.27	0.17	0.43	<0.01
NS	18 (7.6)	18 (4.4)	0.36	0.18	0.71	<0.01
Relation to perpetrator						
Acquaintance	65 (27.4)	94 (23.2)	*Ref.*			
Partner/ex-partner	49 (20.7)	20 (4.9)	0.28	0.15	0.52	<0.01
Contact (met on the day of the SA*)	30 (12.7)	114 (28.1)	2.63	1.58	4.38	<0.01
Virtual contact	43 (18.1)	25 (6.2)	0.40	0.22	0.72	<0.01
No knowledge/recollection/NS	19 (8.0)	82 (20.2)	2.98	1.65	5.39	<0.01
Friend	17 (7.2)	30 (7.4)	1.22	0.62	2.39	0.56
Other*	5 (2.1)	22 (5.4)	3.04	1.10	8.45	0.03
Stranger	9 (3.8)	19 (4.7)	1.46	0.62	3.43	0.39
Crime scene						
Perpetrator’s home	75 (31.6)	90 (22.2)	*Ref.*			
Friend’s home	30 (12.7)	70 (17.2)	1.94	1.15	3.29	0.01
Victim’s home	81 (34.2)	66 (16.3)	0.68	0.43	1.06	0.09
Outdoor location	16 (6.8)	44 (10.8)	2.29	1.20	4.39	0.01
Club/bar	4 (1.7)	27 (6.7)	5.63	1.88	16.8	<0.01
No knowledge/recollection/NS	20 (8.4)	31 (7.6)	1.29	0.68	2.45	0.43
Other**	11 (4.6)	78 (19.2)	5.91	2.93	11.9	<0.01
Number of perpetrators						
1	228 (96.2)	318 (78.3)	*Ref.*			
>1	3 (1.3)	26 (6.4)	6.21	1.86	20.8	<0.01
No knowledge/recollection/NS	6 (2.5)	62 (15.3)	7.41	3.15	17.4	<0.01

NS = Not specified; The information is not specified in the available data, SA = sexual assault

*e.g. family, taxi driver, colleague

**e.g. hotel, car, vacation house, public building

### Type of sexual assault and injuries

In most sexual assaults, the victim did not report any kind of physical violence (59.9%) (Table 3), and we observed no conclusive difference in DFSA compared to non-DFSA (OR 0.71: 95% CI 0.42, 1.19). In 68.3% of sexual assaults, penile penetration was reported, but 26.4% of cases did not remember if such an act took place. The most reported type of penetration was vaginal (73.1%), followed by oral (21.8%) and anal (13.1%), but a large proportion did not remember if such acts took place, 23%, 44.9% and 47.9%, respectively. We observed no conclusive differences in type of sexual assault between DFSA and non-DFSA, however there was an indication of a higher risk of oral penetration in DFSA (OR 1.41: 95% CI 0.92, 2.17). In most cases, there were documented extragenital lesions (86.8%), but no documented anogenital lesions (64.4%). We observed no difference in the prevalence of injuries between DFSA and non-DFSA (Table 3).

**Table 3:** Descriptives of type of sexual assault and objective findings and univariate logistic regression in DFSA compared to non-DFSA

Characteristics	Non-DFSA (*n* (%))	DFSA (*n* (%))	OR	95% C.I. for OR		*p*-value
				Lower	Upper	
Total number	237 (36.9)	406 (63.1)				
Physical violence						
No	36 (15.2)	32 (7.9)	*Ref.*			
Yes	171 (72.2)	214 (52.7)	0.71	0.42	1.19	0.20
No knowledge/recollection/NS	30 (12.7)	160 (39.4)	4.26	2.75	6.61	<0.01
Penile penetration						
No	203 (85.7)	236 (58.1)	*Ref.*			
Yes	15 (6.3)	19 (4.7)	0.92	0.46	1.85	0.81
No knowledge/recollection/NS	19 (8.0)	151 (37.2)	6.27	2.74	14.4	<0.01
Vaginal penetration						
No	212 (89.5)	258 (63.5)	*Ref.*			
Yes	9 (3.8)	16 (3.9)	0.69	0.30	1.58	0.38
No knowledge/recollection/NS	16 (6.8)	132 (32.5)	4.64	1.76	12.2	<0.01
Oral penetration						
No	58 (24.5)	82 (20.2)	*Ref.*			
Yes	107 (45.1)	107 (26.4)	1.41	0.92	2.17	0.11
No knowledge/recollection/NS	72 (30.4)	217 (53.4)	3.01	2.07	4.40	<0.01
Anal penetration						
No	44 (18.6)	40 (9.9)	*Ref.*			
Yes	119 (50.2)	132 (32.5)	0.82	0.50	1.34	0.43
No knowledge/recollection/NS	74 (31.2)	234 (57.6)	2.85	1.99	4.09	<0.01
Extra genital lesions (no/yes)	34 (14.3) / 203 (85.7)	51 (12.6) / 355 (87.4)	1.17	0.73	1.86	0.52
Anogenital lesions (no/yes)	150 (63.3) / 87 (36.7)	264 (65.0) / 142 (35.0)	0.93	0.66	1.30	0.66

NS = Not specified; the information is not specified in the available data

In 416 cases, we had information about the total number of recorded extragenital lesions, most frequently 1–2 lesions were observed (36.5%), but in 22.1% of cases 5 or more lesions were observed (Table 4). According to the forensic records, most lesions were abrasions and bruises on arms and legs and dated to have possibly occurred at the time up to, during, or right after the sexual assault.

**Table 4:** Descriptives of total number of extragenital lesions categorized in 4 groups and univariate logistic regression based on the total number of extragenital lesions

Number of extragenital lesions	Non-DFSA (*n* (%))	DFSA (*n* (%))	OR	95% C.I. for OR		*p*-value
				Lower	Upper	
Total number	144 (34.6)	272 (65.4)				
0	48 (33.3)	76 (27.9)	*Ref.*			
1-2	56 (38.9)	96 (35.3)	1.08	0.66	1.77	0.75
3-5	14 (9.7)	34 (12.5)	1.53	0.75	3.15	0.24
>5	26 (18.1)	66 (24.3)	1.60	0.90	2.86	0.11

We found a tendency towards a higher frequency of many lesions among DFSA cases (> 5 lesions: OR 1.60: 95% CI 0.90–2.86). Similar observations, but with larger p-values, were observed for cases with 3 to 5 lesions (Table 4).

We also performed age stratified analyses (15–24 years and > 24 years) in which we observed that women under the age of 25 were to a larger extent assaulted by someone they had just met, and that penile vaginal penetration were more commonly reported in the age group under the age of 25. In other variables no major discrepancies were observed in the age stratified analyses compared to the analysis in the general population at hand.

#### Time from the sexual assault to examination

Of all victims, 356 cases of sexual assaults were examined within 24 h after the assault, and 272 were examined more than 24 h after (15 not specified) (Table 1). We observed that anogenital injuries were more frequent when the victim was examined within 24 h after the sexual assault (OR 2.60: 95% CI 1.84–3.69) (Table 5). A similar tendency was observed for extragenital lesions.

**Table 5:** Univariate logistic regression of injuries and illicit drugs on all sexual assaults in time from the sexual assault to examination <24 hours compared to >24 hours

	OR	95% C.I. for OR		*p*-value
		Lower	Upper	
Anogenital lesions no/yes	2.60	1.84	3.69	<0.01
Extragenital lesions no/yes	0.80	0.50	1.29	0.36
Extragenital lesions				
0	*Ref.*			
1-2	1.26	0.78	2.02	0.35
3-5	1.43	0.73	2.82	0.30
>5	1.54	0.89	2.68	0.12
Illicit drugs negative/positive	1.42	0.98	2.07	0.07

Table 6 shows injuries and finding of illicit drugs in DFSA compared to non-DFSA, when we only include victims who were examined within 24 hours of the sexual assault. There were no conclusive differences between the groups, but it seems as if DFSA were associated with a tendency for more extragenital lesions, point estimate 1.61 for 1–2 lesions up to a point estimate of 2.05 when observing more than 5 injuries. We also observed conclusively more DFSA victims with positive analysis for illicit drugs compared to non-DFSA (OR 3.03: 95% CI 1.72 – 5.35).

**Table 6:** Univariate logistic regression of injuries and illicit drugs only included victims who were examined <24 hours after the sexual assault on DFSA compared to non-DFSA

	OR	95% C.I. for OR		*p*-value
		Lower	Upper	
Anogenital lesions no/yes	0.99	0.64	1.54	0.98
Extragenital lesions no/yes	1.70	0.88	3.28	0.36
Extragenital lesions				
0	*Ref.*			
1-2	1.61	0.82	3.16	0.17
3-5	1.80	0.69	4.67	0.23
>5	2.05	0.94	4.50	0.07
Illicit drugs negative/positive	3.03	1.72	5.35	<0.01

### Toxicological findings

Out of the 643 cases, 459 were police reported and out of these 123 (26.8%) were analyzed on request from the police, i.e. as part of investigative procedures. In the remaining cases, toxicological analysis was performed as part of the study protocol.

We observed that 307 cases (47.3%) were positive for either drugs or medication with effects on CNS, including sedative antihistamines (Table 7). Of the cases analyzed for ethanol 31.8% was positive (*n* = 130). Of illicit drugs, cocaine was most frequently observed (18.6%, *n* = 116) followed by THC (9.3%, *n* = 60). The most frequently observed medications were antidepressants (15.7%, *n* = 101), followed by benzodiazepines (9.8%, *n* = 63) and antipsychotics (8.8%, *n* = 57). GHB, known colloquially as a prototypical “date rape drug”, was only found in very few cases (< 5), possible due to the drugs very short biological half-life.

**Table 7:** The distribution of the three most frequent found substances, ethanol, cocaine and THC and three most frequent medications, antidepressants, antipsychotics and benzodiazepines

	Total	
	n	%
Total number of cases	643	100
Positive toxicology*	307	47.3
Illicit drugs	158	24.6
Medication	233	36.2
Ethanol		
Total number of cases	409	100
Positive	130	31.8
Negative	279	68.2
Not analyzed**	234	-
THC		
Positive	60	9.3
Negative	583	90.6
Cocaine		
Positive	116	18.6
Negative	527	82.0
Antidepressants		
Positive	101	15.7
Negative	542	84.3
Antipsychotics		
Positive	57	8.8
Negative	586	91.2
Benzodiazepines		
Positive	63	9.8
Negative	580	90.2

*Positive for illicit drugs, medication or both

**The blood samples were not analyzed for ethanol when the examination took place more than 24 hours after the assault

We analyzed toxicological findings for association with DFSA compared to non-DFSA, where we observed a positive association of ethanol with DFSA (4.36, 95% CI 2.51, 7.58) and illicit drugs (OR 2.88, 95% CI 1.88, 4.42). There was no conclusive correlation between DFSA and findings of medication (OR 0.91, 95% CI 0.66–1.27) (Table 8).

**Table 8:** Univariate logistic regression of findings of ethanol, illicit drugs and medication in DFSA cases compared to non-DFSA cases

	OR	95% C.I.for OR		P
		Lower	Upper	
Ethanol				
Negative	*Ref.*			
Positive	4.36	2.51	7.58	<,001
Not analyzed	0.88	0.62	1.25	0.46
Illicit drugs (negative/positive)	2.88	1.88	4.42	<0.01
Medication (negative/positive)	0.91	0.66	1.27	0.60

## Discussion

We observed that female victims of DFSA, compared to non-DFSA, were younger and that assaults more often took place during weekends, at night, and the perpetrator was more often someone that the woman met for the first time on the day of the assault, as in contrast to non-DFSA where it was more often an ex-partner or a contact met in an online fora. In DFSA, it was more common with multiple perpetrators. There was a higher risk of DFSA occurring at a bar or a club, an outdoor location or in the home of a friend. The finding of a larger extent of women below the age of 25 where assaulted by someone they had just met is in contrast to the findings in a previous study about DFSA in victims aged 13–24, that reported a larger amount of cases where the perpetrator was an acquaintance [19] Other studies had the same results as us, but they did not stratify for age [20–24]. It could be speculated that an explanation could be that victims who are assaulted by friends or acquaintances are not reporting the assault or even getting an examination, because they do not want to cause conflict in a friend group. It is also worth considering differences in how acquaintance and stranger are defined. There were no conclusive differences between type of assault, or in observable injuries between DFSA or non-DFSA, but a tendency for an increased risk of oral penetration in DFSA.

The toxicological analyses revealed that ethanol was frequently observed in DFSA and that cocaine and THC were the most frequently detected illicit drugs. Also, the most frequently observed medications were antidepressants, antipsychotics and benzodiazepines. This result was in agreement with a review covering forensic toxicology on global DFSA cases [4] and a previous study in Denmark including only police reported cases [25]. We observed a positive association between both ethanol and illicit drugs with DFSA, but no conclusive correlation between medication and DFSA. We also observed no differences in socioeconomic characteristics, measured as education and low income, between DFSA and non-DFSA victims.

The observation that the risk for DFSA is higher in younger females is in line with most previous research [11, 21, 26–30]. Possible explanations could be a more widespread alcohol culture among young people, a risk prone behavior including experimenting with e.g. illicit drugs and inexperience with the night scene as indicated by assaults during the weekends and nights. Previous studies have linked DFSA to public places [20, 31–33], however one study did not corroborate this association [34], and another study linked DFSA to private home [35]. Of four previous studies [11, 31, 34, 36] only one is in support of our findings of an association between weekends and nights with DFSA [36].

Previous studies have found a positive correlation between extragenital injuries and DFSA [11, 21, 37–40]. We could speculate that the injuries are not related to the sexual assault per se, but because of inebriation with alcohol and/or drugs causing minor accidents such as falls. However, it could also be hypothesized that victims aware of the situations in non-DFSAs could attempt to defend themselves with resultant injuries. In line with this, a study found more lesions following physical force in non-DFSA [41]. Another perspective could be a previous suggestion that victims of SAs that occur outdoors have more injuries inflicted by getting pushed to the ground or against a wall [39].

To our knowledge, no previous study has examined the possible relation between education level, income level and DFSA. Overall, we observed no association between lower socioeconomic position and DFSA compared to non-DFSA victims. We observed that being a high school graduate was associated with a statistically significant 56% increased OR of DFSA compared with having completed only primary school. This finding contradicts the general finding of DFSA victims being younger. However, it should also be noted that educational registry data pertains to the highest level of education attained by the victim at the time of their inclusion in the study, thus many of the victims are in the lower levels due to a relatively young age and the resources to progress to higher levels of education are not captured. Although we did not find any association between low income level and DFSA, we noted that 34.4% of the study population was below 50% of the median national income level (relative poverty), which is in excess of the Danish national average of 4% [42]. However, our measures of socioeconomic position were rather coarse, we suggest future studies to use a finer gradient of income and possible information on social benefits to identify the most vulnerable groups.

Previously, no associations between a mental health condition and risk of DFSA have been observed [43, 44], which is in line with findings of our study. We relied on data based on prescription medication for neuroleptics, antidepressants, mood stabilizers, and anxiolytics, rather than using registries with disease codes. Our approach has the advantage of also identifying patients diagnosed and treated exclusively in the primary sector, e.g. via a family physician or a private psychiatry practice, since Danish registry with diagnosis codes rely on reports from hospitalizations only. Thus, we would argue that using medication as an indicator of disease is a more representative indicator of milder psychiatric diagnoses, e.g. mild or moderate depression. The drawback is that only executed sales are registered.

We did not find more victims of DFSA compared to non-DFSA that were registered as victims of other violent crimes within the last 5 years, or in cases below 18 years of age had reports to the local authorities. According to Statistics Denmark, in 2023 the authorities received reports on 8% of children and adolescents under the age of 18 [45]. In our study population, the prevalence of reports to local authorities within the last five years was 56% among victims of SA. This indicates possibilities for targeted public health interventions to this potentially vulnerable group of adolescents.

Many of the characteristics related to the assault such as day/time, relation to perpetrator, crime scene, intake of alcohol/drugs/medicine originates from self-reported information, and in many cases the victim might have been under the influence of alcohol and/or drugs or maybe in a state of some kind of psychological stress or shock. This is also reflected in the large proportion of missing information in self-reported characteristics (1.1 to 5.6%). Further, some victims might be hesitant to give the correct information about intake of alcohol, medication, or drugs since they could be afraid that it would harm their case if they, voluntarily, have exposed themselves to substantial inebriation.

We found that more anogenital injuries and more extragenital injuries were observed if victims of sexual assault were examined within 24 h after the assault. Similarly, in cases examined within 24 h we observed a higher prevalence of illicit drugs in DFSA compared to non-DFSA. But the risk of selection bias, i.e. that those with more injuries are more prone to report early must be considered as an explanation. Also, the victims exposed to sedating drug effects may also cause a delay in reporting. These findings highlight the importance of an effective course of investigation so that the forensic examination is not unnecessarily delayed and underscores the importance of efforts supporting victims to early reporting of SA, as otherwise there is a risk that important evidence, both in lesions and toxicology, might be missed.

### Strengths and limitations

Our study is based on a large study population of women examined at a center for sexual assault victims in Copenhagen, Denmark. Our findings could be representative of other large western cities. All women were examined by an experienced gynecologist or a forensic physician with expertise in documenting injuries. The examination focused on examining extragenital, genital, and in some cases anal injuries. Furthermore, we linked register-based data with information from large Danish population registers. A strength in this study is the use of both police reported, and non-police reported cases, and the toxicological analysis in almost all cases.

In 416 out of 643 cases the examination was performed by a forensic physician. The examination procedure is standardized, and the gynecologists have been educated to perform the examination by a forensic team. Due to this we do not expect a bias in the examination based on the physician’s specialty, however, this cannot be excluded.

It is a limitation that the time between the sexual assault and the examination varied up to 7 days, as positive toxicology decreases markedly after 12 h [32, 46]. Additionally, there is a tendency for victims of DFSA to consult the CVSA slightly later (> 12 h) than victims of non-DFSA who more often have their medical examination within the first 12 h of the assault [25, 47]. In this study, we were able to discern cases examined within 24 h after the assault, but not specifically identifying those that were examined within 12 h. We only analyzed for ethanol in cases with an examination within 24 h after the sexual assault, thus the proportion of positive cases is less representative. Due to ethanol’s short elimination time, it would be more optimal to analyze samples collected within 12 h. Additionally, we did not find many samples positive for the well-known date rape drugs (e.g. GHB). It is difficult to estimate whether this is due to these substances not being used or because these drugs are simply not detected due to their short half-life.

We found that 63.1% of all included cases were categorized as alleged DFSA, which may seem like a large proportion, suggesting too wide DFSA inclusion criteria (Fig. 2). However, the result is similar to observations in a previous study [25] including both opportunistic and proactive DFSA, and we observed positive toxicology in about half of the DFSA-cases (not including ethanol). Thus, we consider the applied inclusion criteria relevant though the true prevalence of DFSA and especially proactive DFSA is difficult to verify.

Since the time between the assault and the examination could vary, injuries might have healed, and drugs or medication might have been metabolized at the time of the examination. We speculate that information on other socioeconomic variables might have provided more nuanced pictures of socioeconomic differences between the groups, as such we recommend expanding the analyses on differences in income between the two groups of sexual assaults. As many of the variables were dependent upon the reporting of the victim, these were flawed since a large proportion could, as an example, not remember the type of sexual assault. To overcome this obstacle, perhaps findings from the later part of the police investigation can provide further information. In our study, the majority of cases were females and there were too few cases of men and transgender persons to allow for meaningful statistical analysis. It is possible that the characteristics of DFSA in the group of men and transgender individuals differs from those of females, which future studies should examine. The low prevalence of male victims and victims identifying as other genders is in line with previous results [12]. We speculate this may partially be due to an overrepresentation of females among victims of sexual abuse [48].

## Conclusion

Female victims of DFSA were younger compared to non-DFSA, and DFSA more frequently occurred during weekends, at night and by a perpetrator that the woman had met for the first time on the day of the assault. Additionally, DFSA was associated with multiple perpetrators. There was a higher risk for DFSA in bars or clubs, but also for in an outdoor location or in the home of a third party. There were no conclusive differences between type of assault or in observable injuries between DFSA or non-DFSA, but a tendency for an increased risk of oral penetration in DFSA. The toxicological analyses revealed that ethanol was frequently encountered in DFSA, and the most frequently detected illicit drugs were cocaine and THC. The most frequently observed medications were antidepressants, antipsychotics, and benzodiazepines. We observed no differences in socioeconomic characteristics measured as education and low income between DFSA and non-DFSA victims. We observed that victims of sexual assault under the age of 18 seem to be a vulnerable group with more reports to child protection services compared to the general population. The indication that sexual assault cases examined within 24 h after the sexual assault had more anogenital lesions and extragenital lesions compared to those examined later, highlights the importance of prioritizing the forensic examination not only for securing samples for toxicology but also for documenting injuries.
